# Silencing of ANGPTL 3 (angiopoietin-like protein 3) in human hepatocytes results in decreased expression of gluconeogenic genes and reduced triacylglycerol-rich VLDL secretion upon insulin stimulation

**DOI:** 10.1042/BSR20140115

**Published:** 2014-12-12

**Authors:** Anna Tikka, Jarkko Soronen, Pirkka-Pekka Laurila, Jari Metso, Christian Ehnholm, Matti Jauhiainen

**Affiliations:** *National Institute for Health and Welfare, Public Health Genomics Unit, Biomedicum, Helsinki, Finland; †Minerva Foundation Institute for Medical Research, Helsinki, Finland

**Keywords:** ANGPTL3 silencing, hypolipidaemia, insulin signalling, liver, rosiglitazone, VLDL, ANGPTL, angiopoietin-like protein, CCD, coiled coil domain, FHBL2, familial combined hypolipidaemia, GLUT2, glucose transporter 2, HDL, high-density lipoprotein, IHH, immortalized human hepatocyte, IR, insulin receptor, IRS, insulin receptor substrate, LDL, low-density lipoprotein, LPL, lipoprotein lipase, NEFA, non-esterified fatty acid, PEPCK, phosphoenolpyruvate carboxykinase, PGC1α, peroxisome proliferator-activated receptor γ co-activator 1-α, PI3K, phosphoinositide 3-kinase, PL, phospholipid, PPAR, peroxisome-proliferator-activated receptor, QPCR, quantitative PCR, shRNA, small hairpin RNA, TAG, triacylglycerol, TRB3, tribbles homologue 3, VLDL, very-low-density lipoprotein

## Abstract

Homozygosity of loss-of-function mutations in *ANGPTL3* (angiopoietin-like protein 3)-gene results in FHBL2 (familial combined hypolipidaemia, OMIM #605019) characterized by the reduction of all major plasma lipoprotein classes, which includes VLDL (very-low-density lipoprotein), LDL (low-density lipoprotein), HDL (high-density lipoprotein) and low circulating NEFAs (non-esterified fatty acids), glucose and insulin levels. Thus complete lack of ANGPTL3 in humans not only affects lipid metabolism, but also affects whole-body insulin and glucose balance. We used wild-type and *ANGPTL3-*silenced IHHs (human immortalized hepatocytes) to investigate the effect of *ANGPTL3* silencing on hepatocyte-specific VLDL secretion and glucose uptake. We demonstrate that both insulin and PPARγ (peroxisome-proliferator-activated receptor γ) agonist rosiglitazone down-regulate the secretion of ANGPTL3 and TAG (triacylglycerol)-enriched VLDL1-type particles in a dose-dependent manner. Silencing of *ANGPTL3* improved glucose uptake in hepatocytes by 20–50% and influenced down-regulation of gluconeogenic genes, suggesting that silencing of *ANGPTL3* improves insulin sensitivity. We further show that *ANGPTL3*-silenced cells display a more pronounced shift from the secretion of TAG-enriched VLDL1-type particles to secretion of lipid poor VLDL2-type particles during insulin stimulation. These data suggest liver-specific mechanisms involved in the reported insulin-sensitive phenotype of ANGPTL3-deficient humans, featuring lower plasma insulin and glucose levels.

## Abstract 2

We show that silencing of ANGPTL3 in human hepatocytes in addition to reducing secretion of TAG-enriched VLDL upon insulin stimulation enhances glucose uptake and improves insulin response. Thus, our data provide insight into the lower insulin and glucose levels observed in humans with ANGPTL3 loss-of-function mutation.

## INTRODUCTION

The ANGPTLs (angiopoietin-like proteins) are a family of secreted proteins which share tertiary structural domains with angiopoietins [1], with N-terminal CCD (coiled coil domain) and C-terminal FLD (fibrinogen-like domain) of which the CCD is functionally associated with reduced plasma TAGs (triacylglycerols) [2]. *ANGPTL3* is almost exclusively expressed in the liver with minor expression found in kidney [3,4]. ANGPTL3 has been shown to inhibit LPL (lipoprotein lipase) *in vitro* and *in vivo* [5–7], and mice lacking ANGPTL3 protein have increased LPL activity and reduced levels of plasma TAG [8,9].

GWAS (genome wide association studies) in humans have shown that SNPs (single nucleotide polymorphisms) at loci near *ANGPTL3* are associated with plasma TAG levels [10–12]. Sequencing of the coding region of *ANGPTL3* revealed additional loss-of-function mutations associated with low levels of plasma TAG [4]. Furthermore, Musunuru et al. [13] have identified E128X and S17X as two novel nonsense mutations in *ANGPTL3.* Loss of function in *ANGPTL3* results in a condition referred to as FHBL2 (familial combined hypolipidaemia, OMIM #605019), featuring reduction in all major plasma lipoproteins VLDL (very-low-density lipoprotein), LDL (low-density lipoprotein) and HDL (high-density lipoprotein). In addition to FHBL2 phenotype also plasma NEFAs (non-esterified fatty acids), insulin, fasting plasma glucose and HOMA-IR (homoeostatic model assessment of insulin resistance) values are significantly lower in individuals homozygous for S17X mutation compared with carriers and non-carriers [14,15] linking the hypolipidaemic effect of ANGPTL3-deficiency with insulin sensitivity.

Insulin is the primary hormone controlling whole body glucose and lipid homoeostasis. Activation of the insulin-signalling pathway suppresses hepatic secretion of TAG-enriched VLDL1 particles without affecting the secretion of lipid-poor VLDL2 [16]. The number of TAG-enriched VLDL1 particles is elevated during insulin resistance, contributing to hypertriglyceridaemia [17]. Despite elevated plasma glucose and TAG levels liver persistently continues glucose production and VLDL secretion during IR [18,19]. Insulin inhibits VLDL secretion through PI3K (phosphoinositide 3-kinase)-mediated abruption in the second-step bulky TAG-assembly of VLDL1 particles and apoB-100 availability [20]. Unlike in the muscle and adipose tissue, insulin does not directly increase glucose uptake in the liver. Postic et al. [21] demonstrated down-regulation of GLUT2 (glucose transporter 2) by insulin in a dose-dependent manner *in vivo* and *in vitro*. Hepatic GLUT2 expression is induced by high glucose concentration which, during feeding, prevent the inhibitory effect of insulin on GLUT2 expression [21].

We utilized wild-type IHHs (immortalized human hepatocytes) and IHH cells with silenced ANGPTL3 to study how the secretion levels of ANGPTL3 are regulated in hepatocytes and how the silencing of *ANGPTL3* affects VLDL secretion and hepatocyte-specific glucose uptake. The aim of the research is to seek further insight into the molecular mechanisms related to hypolipidaemia and insulin sensitivity reported in humans with ANGPTL3 deficiency [14].

## MATERIALS AND METHODS

### *ANGPTL3*-silenced cell-line and cell culture conditions

Liver IHHs immortalized by SV40 large T-Antigen (IHH, ATCC® PTA-5565™) were transduced with MISSION™ shRNA (small hairpin RNA) Lentiviral Vector particles (TRCN0000242782, Sigma Aldrich) targeting *ANGPTL3* (NM_014495.2) or with non-target shRNA (SHCOO2, Sigma Aldrich) [MOI (multiplicity of infection) 1]. Positive cells were selected against 5 μg/ml puromycin for 12 days. The transduction was repeated once after the first selection. Cells were cultured in Williams medium E (Gibco by Life Technologies, 22551-022) with added 10% (v/v) FBS and glutamine 0.2 mg/ml and incubated +37 °C. FBS was removed during experiments and total protein from cell lysates was used to normalize the data. Cells were washed with PBS (pH 7.4) and lysed in RIPA buffer. Protein concentration was measured with Bradford protein assay (Bio-Rad). Following compounds were used: insulin (bovine, Sigma-Aldrich), Wortmannin (Sigma-Aldrich), Akt1/2 inhibitor (Sigma-Aldrich), rosiglitazone (Cayman Chemical) and GW9662 (Sigma-Aldrich).

### Enzymatic measurements of TAG and PLs (phospholipids)

TAGs from media and cell lysates were measured with an enzymatic method (GPO-PAP 1488872 kit; Roche Diagnostics Gmbh). Phospholipids were measured from the cell culture media with an enzymatic method (FS 60080970; Diasys Diagnostics).

### ANGPTL3 and apoB-100 ELISA assays

Concentration of ANGPTL3 was measured utilizing ELISA assay developed by Robciuc et al. [22]. ApoB-100 was measured with Human apolipoprotein B ELISA kit (Mabtech) according to instructions provided by the manufacturer.

### Labelled oleic acid and TAG extraction

Cells were incubated in the presence of 5.5 mM glucose and 0.5% (w/v) BSA-complexed with 0.375 mM oleic acid and 0.1 μCi [^14^C]-oleic acid (PerkinElmer) for 24 h. Cells were collected in 1 ml 2% NaCl–PBS buffer. Lipids were extracted by adding 2 ml of methanol and 1 ml chloroform and centrifuged (2500 rev/min for 10 min at room temperature). H_2_O (1 ml) and chloroform (1 ml) were added and again centrifuged (2500 rev/min, for 10 min at room temperature). The upper phase was removed and the lower phase dried under nitrogen and solubilized in 100 μl of chloroform and applied on a TLC-plate. The TLC-plates were run in a chamber containing (*N*-hexane, diethylether, acetic acid and H_2_O (65/15/1/0.25, v/v). Iodine vapour stained areas containing the TAGs were scraped out from the TLC-plate and radioactivity was measured by liquid scintillation counting (Wallac LS-Beta-Counter).

### Measurement of glucose uptake

Cells were incubated in the presence of 5.5 or 20 mM glucose and 1 μCi of deoxy-D-glucose, 2-[1,2-^3^H (N)] (1 mCi/ml, lot 638084, Perkin Elmer). Radioactivity from cell lysates and media was measured by liquid scintillation counter (Wallac LS-Beta-Counter).

### QPCR (quantitative PCR) analysis

RNA was extracted with Qiagen RNA purification kit and synthesized with SuperScript® VILO™ cDNA Synthesis Kit (Life Technologies). Each QPCR contained primers (see Table 1) at 300 nM concentration and 10 ng of cDNA. The total volume of each reaction was 10 μl containing SYBR® Green PCR Master Mix (Life Technologies). The QPCR program: 95 °C for 10 min followed by 45 cycles 95 °C 15 s, 60 °C 1 min.

**Table 1:** QPCR-primers

Name, gene name	Forward	Reverse
*ACTIN*, *ACTB*	GATGTGGATCAGCAAGCAGGA	AGCATTTGCGGTGGACGAT
*ANGPTL3*	CCAGAACACCCAGAAGTAACT	TCTGTGGGTTCTTGAATACTAGTC
*ANGPTL4*	GCCTATAGCCTGCAGCTCAC	CAAGTGGAGAAGGGTACGGA
*ANGPTL8*, *C19ORF80*	AGGTCTTAAAGGCTCACG	TTCCATCCAGGCAGATTC
*DGAT2*	CATCCTCATGTACATATTCTGC	TGGGAAAGTAGTCTCGAAAG
*HNF4α*, *HNF4A*	AGTACATCCCAGCTTTCTG	AATGTAGTCATTGCCTAGGAG
*IL-6*	GGTACATCCTCGACGGCATCT	GTGCCTCTTTGCTGCTTTCAC
*IRS-1*	CAGAATGAAGACCTAAATGACC	ATGCATCGTACCATCTACTG
*IRS-2*	AAGAGTGAAGATCTGTCTGG	ATCTAACAGAGTCCACAGATG
*PEPCK*, *PCK1*	GGGCATCCTCAGGCGGCT	GATAACCGTCTTGCTTTCGAT
*PGC1A*, *PPARGC1A*	GCAGACCTAGATTCAAACTC	CATCCCTCTGTCATCCTC
*PI3K 85*, *PIK3R1*	AAGAAGACTTGAAGAAGCA	TCAACCACATCAAGTATTGG
*PPARALPHA*, *PPARA*	CCTAAAAAGCCTAAGGAAACC	GATCTCCACAGCAAATGATAG
*PPAR*γ, *PPARG*	GATCCAGTGGTTGCAGATTACAA	GAGGGAGTTGGAAGGCTCTTC
*PTEN*	GGCTAAGTGAAGATGACAATC	GTTACTCCCTTTTTGTCTCTG
*TGH*, *TGH/CES1*	GTCTTTCTGGGCATTCCATT	CTCTCCACGTCTTGTAGGCA
*TNFα*, *TNFA*	CCCAGGCAGTCAGATCATCTT	AGCTGCCCCTGAGCTTGA
*TRB3*, *TRIB3*	GACCGTGAGAGGAAGAAG	GAGTATCTCAGGTCCCAC

### Statistics

The experiments were conducted in triplicate (*n*=3) and performed using 2–3 separately transduced IHH cell cultures. Results are expressed as means±S.D. One-way or two-way ANOVA was used to compare the significance between groups followed by Games–Howell *post hoc* test. The difference between groups was considered statistically significant if *P*<0.05 using Games–Howell *post hoc* test (*<0.05, **<0.01, ***<0.001).

## RESULTS

**Figure 1:**
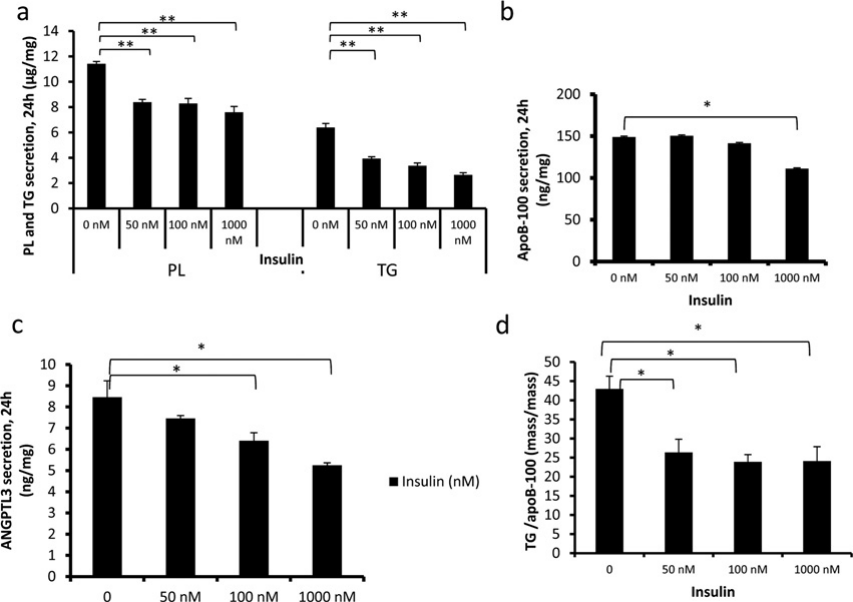
The effect of insulin on VLDL and ANGPTL3 secretion Wild-type IHH-cells were incubated in 5.5 mM glucose for 24 h at variable concentrations of insulin. (**a**) Secretion of TAG and PL during insulin stimulation (μg/mg). (**b**) Secretion of apoB-100 (ng/mg). (**c**) Secretion of ANGPTL3 (ng/mg). (**d**) The amount of TAG (ng) for each ng of apoB-100.

We first tested insulin response in wild-type IHH cells by incubating the cells in the presence of insulin and measured the secretion rate of VLDL by analysing the cell culture media for TAG, apoB-100 and PLs after 24 h. Insulin treatment resulted in a dose dependent down-regulation of the secreted TAG, PL (Figure 1a) and apoB-100 (Figure 1b). Insulin in a dose-dependent manner reduced also the secretion of ANGPTL3 (Figure 1c). We calculated a TAG/apoB-100 ratio and demonstrated that insulin stimulation results in a significant reduction of hepatocyte TAG secreted per apoB-100 protein (Figure 1d). These data are in line with reports on insulin mediated down-regulation of TAG-enriched VLDL1 particles without affecting the (lipid-poor) VLDL2 secretion [16,23].

**Figure 2:**
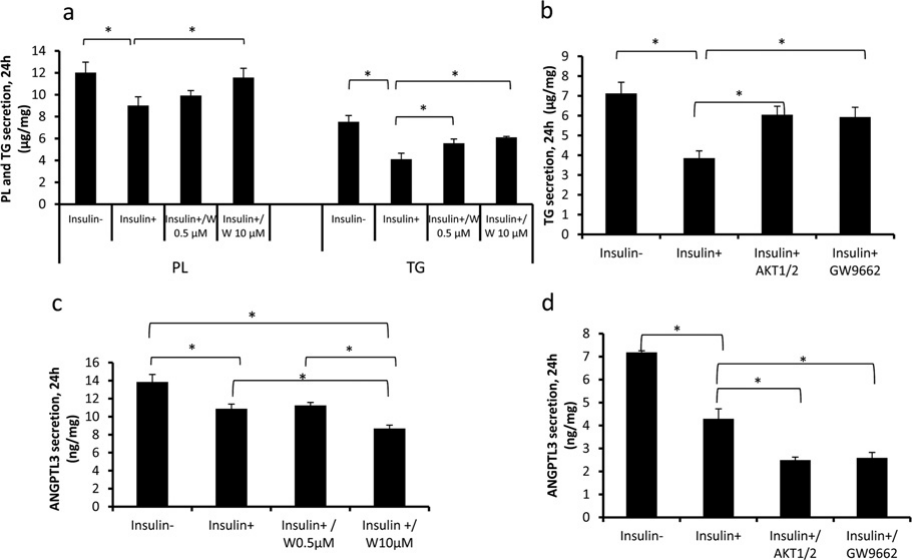
The effect of PI3K Wortmannin, Akt-specific inhibitor Akt1/2 and PPARγ antagonist GW9662 on VLDL and ANGPTL3 secretion Wild-type IHH-cells were incubated in 5.5 mM glucose and 100 nM insulin (−/+), Wortmannin (0.5 μM,10 μM), Akt1/2 inhibitor (20 μM) or PPARγ antagonist GW9662 (10 μM) for 24 h. (**a**) Secretion of TAG and PL (μg/mg). (**b**) Secretion of TAG (μg/mg). (**c**) Secretion of ANGPTL3 (ng/mg). (**d**) Secretion of ANGPTL3 (ng/mg).

Insulin mediates its functions via IRS (insulin receptor substrate)–PI3K-dependent pathway [24]. To test whether VLDL secretion in IHH cells is regulated via PI3K and its downstream target Akt, we incubated cells with a PI3K inhibitor Wortmannin and Akt1/2 inhibitor. Treatment with Wortmannin partly abolished the inhibitory effect of insulin on TAG and PL secretion (Figure 2a). Akt1/2 inhibitor, much like Wortmannin, partially abolished the inhibitory effect of insulin on TAG secretion indicating that insulin regulates the secretion of VLDL via PI3K–Akt pathway in IHH cells (Figure 2b). Wortmannin or Akt1/2 did not abolish the inhibitory effect of insulin on ANGPTL3 secretion suggesting that secretion of ANGPTL3 is not down-regulated via PI3K or Akt (Figures 2c and 2d).

**Figure 3:**
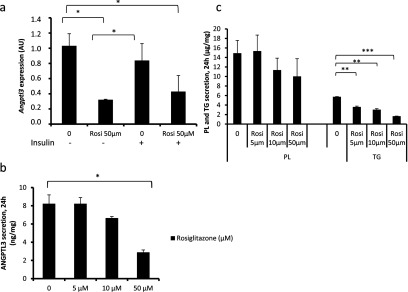
The effect of rosiglitazone on VLDL and ANGPTL3 secretion Wild-type IHH-cells were incubated in 5.5 mM glucose and 100 nM insulin (+/−) for 24 h following RNA extraction from cell lysates. Secretion of ANGPTL3 PL and TAG was measured in the presence of rosiglitazone. (**a**) Expression of *ANGPTL3* (AU, arbitary unit). (**b**) Secretion of ANGPTL3 (ng/mg). (**c**) Secretion of TAG and PL (μg/mg).

Since ANGPTL3 protein secretion is regulated by insulin, we investigated whether nuclear PPARs (peroxisome proliferator-activated receptors) α and γ and their agonists fenofibrate, used to treat high plasma TAG and cholesterol levels, and rosiglitazone, an insulin sensitizer, would affect the secretion of ANGPTL3. We demonstrate that PPARγ agonist rosiglitazone, in a dose-dependent manner, down-regulated mRNA expression (Figure 3a) and the secretion of ANGPTL3 (Figure 3b). PPARα agonist fenofibrate did not affect the secretion of ANGPTL3 (results not shown). Rosiglitazone, much like insulin, reduced the secretion of TAG in a dose-dependent manner (Figure 3c). PPARγ antagonist (GW9662) abolished the inhibitory effect of insulin on TAG secretion (Figure 2b). These data demonstrate that rosiglitazone mimics insulin action in cultured human hepatocytes by decreasing the secretion of TAG and ANGPTL3 in a dose-dependent manner. These data also support the hypothesis that insulin may down-regulate VLDL secretion via PPARγ. However, PPARγ-antagonist (GW9662) did not abolish the inhibitory effect of insulin on ANGPTL3 secretion (Figure 2d) indicating that insulin and PPARγ down-regulate the expression of ANGPTL3 via different pathways.

**Figure 4:**
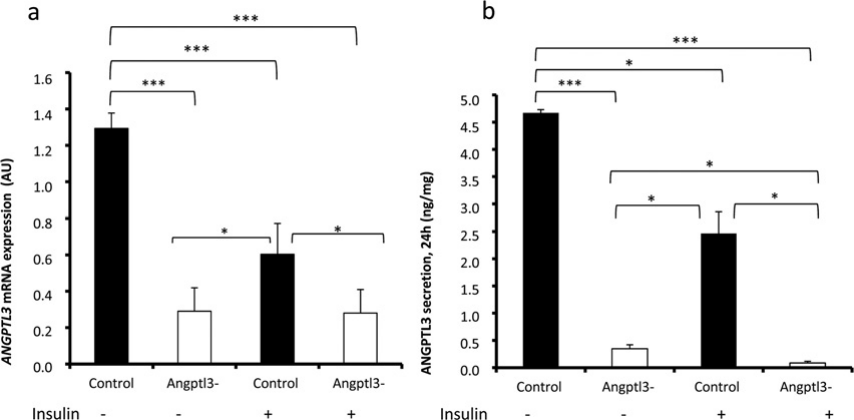
Silencing of *ANGPTL3* IHH-cells were transduced with *ANGPTL3*-targeting or non-targeting shRNA (control). Cells were incubated in 5.5 mM glucose with 100 nM insulin (+/−) for 24 h. RNA was extracted from cell lysates. (**a**) Expression of *ANGPTL3* (AU, arbitrary unit). (**b**) Secretion of ANGPTL3 (ng/mg).

**Figure 5:**
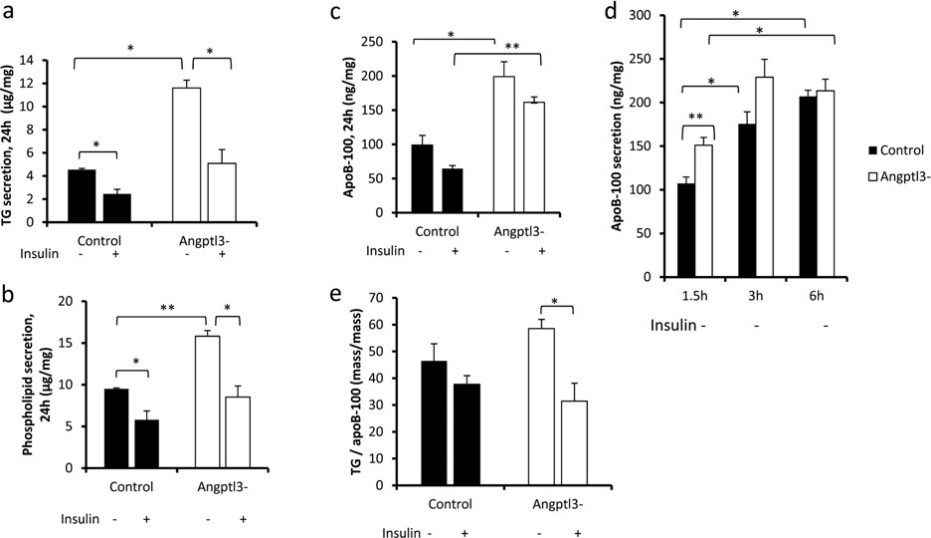
Secretion of VLDL from *ANGPTL3*-silenced and control IHH cells *ANGPTL3*-silenced and control cells were incubated in media containing 5.5 mM glucose and 100 nM insulin (+/−). (**a**–**c**) Secretion of TAG, PL (μg/mg) and apoB-100 (ng/mg). (**d**) Secretion of short-term apoB-100 (ng/mg). (**e**) The amount of TAG (ng) for each ng of apoB-100.

To investigate ANGPTL3-specific functions in hepatocytes, we silenced *ANGPTL3*-gene by transducing wild-type IHH cells with a lentiviral vector carrying *ANGPTL3*-targeting shRNA-fragment. Control cells were transduced with non-targeting shRNA. We achieved 85% silencing in *ANGPTL3* expression (Figure 4a) and a 90–95% decrease in ANGPTL3 protein secretion (Figure 4b). We then measured the concentration of TAG, PL and apoB-100 from the cell culture media after 24 h incubation. Surprisingly the secretion of apoB-100, PL and TAG were all increased in *ANGPTL3*-silenced cells compared with control cells under non-insulin conditions (−) (Figures 5a–5c). Also a short-term secretion rate of apoB-100 was higher in *ANGPTL3*-silenced cells under non-insulin conditions (Figure 5d). Insulin stimulation (+) reduced the secretion of TAG and PL in silenced cells (Figures 5a–5b) near to the level of TAG secreted by the control cells with a smaller decrease in secreted apoB-100 levels (Figure 5c). We calculated a TAG/apoB-100 ratio for both control and silenced cells and concluded that the ratio was more dramatically reduced in *ANGPTL3*-silenced cells [47%, from 60 to 32 (mass/mass)] compared with control cells [16%, from 45 to 38 (mass/mass)] during insulin stimulation (+) (Figure 5e). These findings suggest that the silencing of *ANGPTL3* results in a more pronounced shift from the secretion of large TAG-enriched VLDL1-type particles into small lipid-poor VLDL2-type particles during insulin stimulation. These data are in line with plasma measures from ANGPTL3-deficient subjects who had a 4.3-fold reduction in the TAG/apoB-100 ratio in VLDL when compared with non-carriers [14] and suggests that *ANGPTL3*-silenced cells display enhanced insulin sensitivity as VLDL1 particles dominate the VLDL profile in insulin-resistant states.

**Figure 6:**
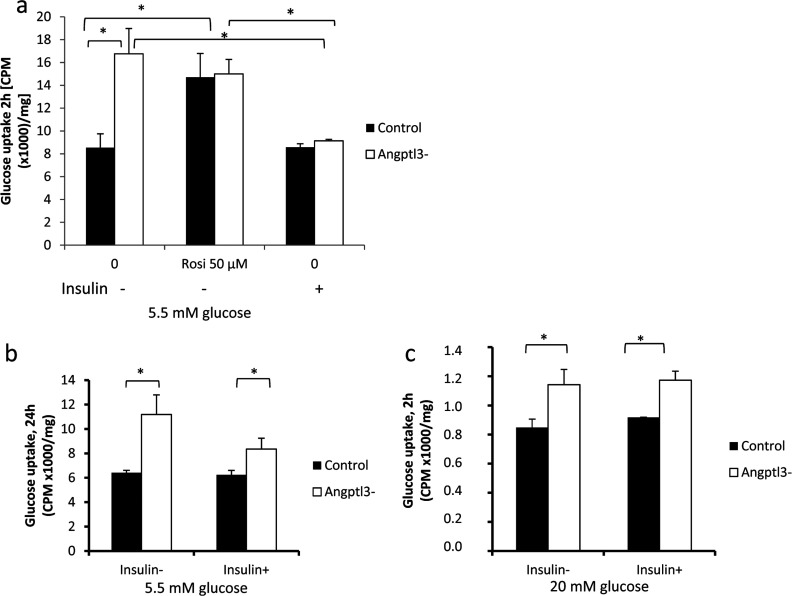
Hepatic glucose uptake in *ANGPTL3*-silenced and control cells *ANGPTL3*-silenced and control cells were incubated in the presence of [^3^H] labelled deoxy-D-glucose. Radioactivity (CPM) was measured from the cell lysates. (**a**) Hepatic glucose uptake measured after 2 h incubation period in media containing 5.5 mM glucose (CPM/mg). Cells were incubated with 50 μM rosiglitazone or 100 nM insulin (+/−). (**b**) Hepatic glucose uptake measured after 24 h incubation period in media containing 5.5 mM glucose and 100 nM insulin (+/−) (CPM/mg). (**c**) Hepatic glucose uptake measured after 2 h incubation period in media containing 20 mM glucose and 100 nM insulin (+/−) (CPM/mg).

We then measured glucose uptake in control and silenced cells by utilizing a non-hydrolysable radiotracer, [^3^H]-labelled deoxy-D-glucose. Basal glucose uptake without insulin stimulus was increased by 50% in *ANGPTL3* silenced cells compared with control cells after 2 h (Figure 6a) and after 24 h (Figure 6b). Treatment with rosiglitazone improved hepatocyte glucose uptake by 40% in control cells (Figure 6a). Acute insulin stimulus in the presence of 5.5 mM glucose did not increase hepatocyte glucose uptake in control cells and significantly decreased glucose uptake in *ANGPTL3*-silenced cells after 2 h (Figure 6a) and 24 h (Figure 6b). When the glucose concentration was increased up to 20 mM the inhibitory effect of insulin on glucose uptake was abolished (Figure 6c). We conclude that the silencing of *ANGPTL3* results in increased glucose uptake comparable with rosiglitazone treatment in the absence of insulin stimulus and that insulin inhibits hepatocyte-specific glucose uptake in the presence of low glucose concentrations but not under high glucose concentration.

**Figure 7:**
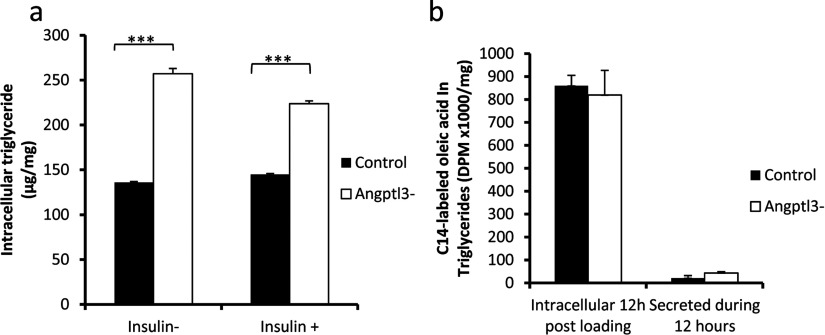
Intracellular TAG and fatty acid uptake in *ANGPTL3*-silenced and control cells (**a**) Intracellular TAGs were measured in *ANGPTL3*-silenced and control cell lysates after 24 h incubation with 5.5 mM glucose and 100 nM insulin (+/−). (**b**) *ANGPTL3*-silenced and control cells were incubated in media containing 5.5 mM glucose, 0.375 mM oleic acid complexed with 0.5% BSA and C^14−^ labelled oleic acid for 12 h. Radioactivity (DPM) in intracellular TAG and secreted TAG was measured (DPM/mg).

We reasoned that the increased glucose uptake in *ANGPTL3*-silenced hepatocytes would cause elevation in glycogen and intracellular TAG levels and measured the intracellular TAG levels after 24 h incubation period. We observed elevated intracellular TAG levels in *ANGPTL3*-silenced cells as compared with control cells (Figure 7a). To test whether silencing of *ANGPTL3* affected hepatic fatty acid uptake we incubated control and *ANGPTL3*-silenced cells in media containing [^14^C]-labelled oleic acid bound to 0.5% BSA for 12 h and measured the uptake of fatty acids and its conversion into intracellular TAG. There were no differences in fatty acid incorporation in intracellular TAG and the secretion rates of TAG between control and *ANGPTL3*-silenced cells (Figure 7b).

**Figure 8:**
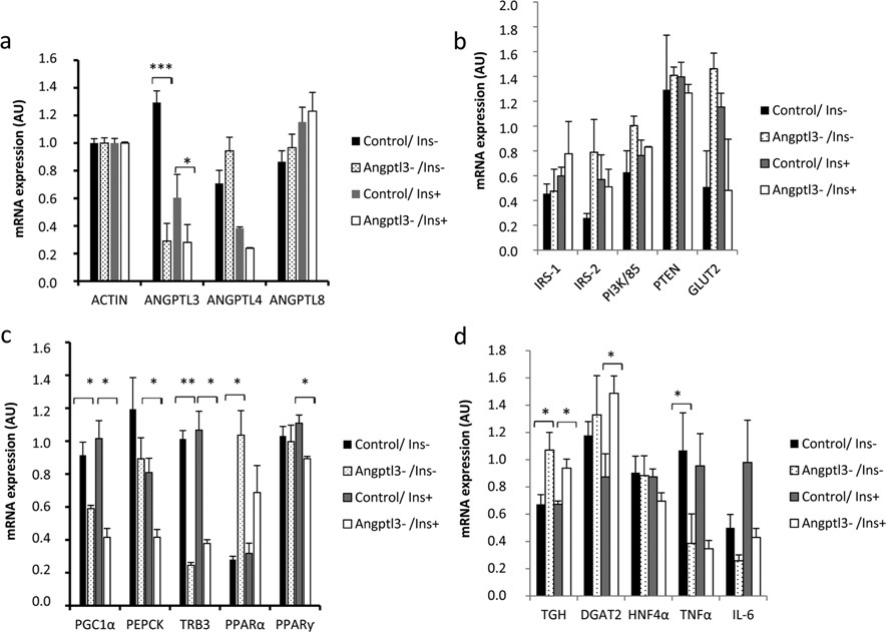
Gene expression patterns in *ANGPTL3*-silenced and control IHH cells during non-insulin condition (Ins-) and insulin stimulation (Ins+) (**a–d**) *ANGPTL3*-silenced cells and control cells were incubated with 5.5 mM glucose and 100 nM insulin (+/−) for 24 h followed by total RNA extraction from cell lysates, cDNA synthesis and q-PCR (AU, arbitrary unit).

Finally we investigated whether silencing of *ANGPTL3* changes the expression of insulin responsive genes. QPCR demonstrated that there were no significant changes in gene expression levels of *ANGPTL8* or *ANGPTL4* between control and silenced cells (Figure 8a). Insulin receptor *IRS-2*, *GLUT2* and *PI3K* (regulatory subunit p85) show a moderate (but non-significant) up-regulation in *ANGPTL3*-silenced cells in non-insulin conditions without any differences in *IRS-1* or PI3K inhibitor PTEN (phosphatase and tensin homologue deleted on chromosome 10) (Figure 8b). Expression of *PGC1α* (peroxisome proliferator-activated receptor γ co-activator 1-α) and its downstream targets *PEPCK* (phosphoenolpyruvate carboxy-kinase) and *TRB-3* (tribbles homologue 3) were significantly down-regulated in *ANGPTL3*-silenced cells, whereas expression of *PPARα* was significantly up-regulated in *ANGPTL3*-silenced cells (Figure 8c). The mRNA levels of PPARγ displayed a moderate down-regulation in *ANGPTL3*-silenced cells upon insulin stimulation (Figure 8c). The expression of TGH (TAG hydrolase) and DGAT2 (diacylgylcerol acyltransferase 2) involved in VLDL assembly were up-regulated in *ANGPTL3*-silenced cells during insulin stimulus while the expression of inflammation marker TNFα (tumour necrosis factor α) was significantly down-regulated in the silenced cells (Figure 8d). There was no significant difference in IL-6 (interleukin-6) or HNF4α (hepatocyte nuclear factor α) expression (Figure 8d). These data suggest a down-regulation of gluconeogenic genes and an increase in genes promoting TAG synthesis in *ANGPTL3*-silenced cells as compared with control.

## DISCUSSION

Since ANGPTL3 is primarily expressed in the liver [3,4] and affects TAG clearance [5–9] and possibly fatty acid release from adipose tissue [25] it is unclear how extracellular signals affect ANGPTL3 secretion rates and how silencing of *ANGPTL3* would affect glucose and lipid metabolism in the liver. We show that activation of PPARγ by its agonist rosiglitazone, much like insulin, decreased the expression and secretion of ANGPTL3 accompanied by reduced secretion of TAG. The association of rosiglitazone and reduced VLDL–TAG secretion has been established *in vivo* [26,27]. Down-regulation of *ANGPTL3* gene expression upon rosiglitazone treatment can also be found in microarray data of primary human hepatocytes [28] and is verified by our data including decreased dose-dependent protein secretion. The effect of insulin on *ANGPTL3* expression and secretion was shown in HepG2 cells [29] and also verified by our data. These findings indicate that *ANGPTL3* is a target gene for insulin and PPARγ action and demonstrate that stimulation of PPARγ by rosiglitazone, mediates or mimics insulin action with a concomitant reduction in ANGPTL3 and VLDL secretion.

Insulin stimulation reduces the secretion of TAG-enriched VLDL1-type particles in the liver without affecting the basal secretion of the lipid-poor VLDL2-type particles [16]. This balance is disrupted in IR leading to increased secretion of VLDL1 and increased plasma lipid levels [16,17]. Interestingly a loss of function mutation in the *ANGPTL3-*gene results in decreased levels of all lipoprotein types in plasma [14] but whether the hypolipidaemic phenotype is caused by decreased hepatic VLDL secretion or due to enhanced TAG clearance in the peripheral tissue (or both) has not been verified clearly. The work by Musunuru et al. [13] suggested lower apoB-100 production rate in homozygous human carriers of ANGPTL3 loss of function mutations. Shimizugawa et al. [9] and Ando et al. [30] suggest that the low plasma lipid levels (especially TAGs) observed in ANGPTL3-deficient mice were likely caused by increased VLDL–TAG clearance via induced LPL activity rather than decreased VLDL secretion.

We did not detect decreased apoB-secretion in *ANGPTL3*-silenced cells compared with control; however, silencing of *ANGPTL3* caused a major decrease in secreted TAG during insulin stimulation with a more subtle effect on apoB-100 secretion. This observation is in line with the analysis on VLDL particles isolated from plasma of *ANGPTL3*-deficient subjects showing a dramatic reduction in VLDL particle size and a 4.3-fold reduction in the TAG/apoB-100 ratio [14]. We demonstrate that a shift from the secretion of TAG-enriched VLDL1-type particles into the secretion of TAG poor VLDL2-type particles under insulin stimulus was more pronounced in ANGPTL3 silenced cells compared with control cells suggesting elevated insulin sensitivity. Higher insulin sensitivity in homozygous *ANGPTL3* loss-of-function carriers was demonstrated previously by Robciuc et al. [14] thus supporting our cell data. Additionally Inukai et al. [31] showed elevated ANGPTL3 expression in mice in both insulin-deficient and -resistant diabetic states. Therefore down-regulation of *ANGPTL3* might have a protective role against IR.

Liver uptake accounts for about 30% disposal of plasma glucose [32]. Silencing of *ANGPTL3* resulted in 20–50% increase in glucose uptake compared with control cells and probably explaining glucose conversion into intracellular TAGs and their increased secretion in *ANGPTL3*-silenced cells under insulin-depleted conditions. Human data do not support an increased prevalence of hepatic steatosis in *ANGPTL3*-deficient subjects [15,33] suggesting that fluctuations in the availability of substrates, plasma glucose and insulin concentrations *in vivo* as well as uptake of glucose and fatty acids by other tissues regulate hepatic glucose uptake and VLDL secretion and thereby preventing hepatic TAG accumulation in ANGPTL3-deficient individuals.

**Figure 9:**
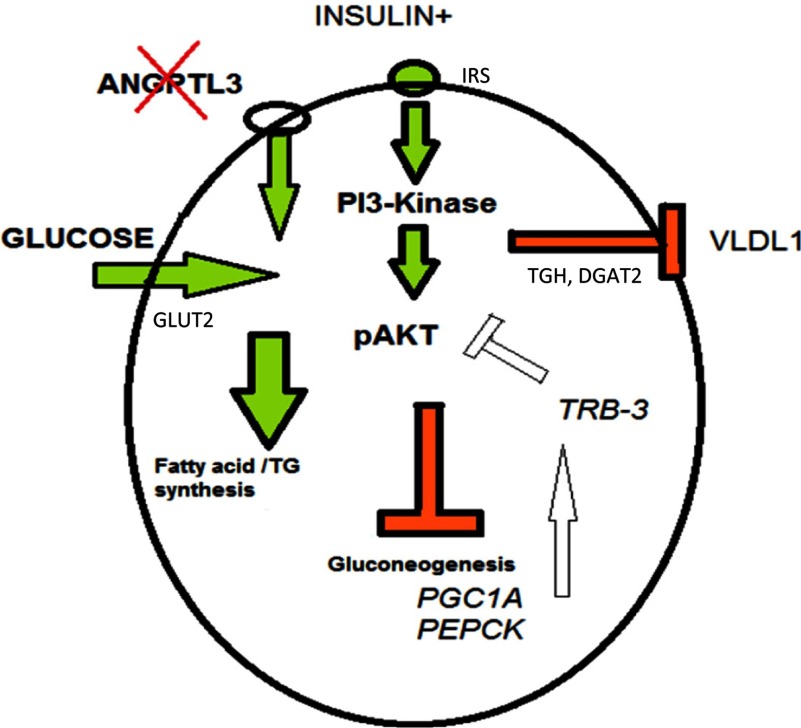
Hypothetic model for ANGPTL3 function in a hepatocyte Silencing of ANGPTL3 enhances GLUT2-mediated glucose uptake, increases TAG synthesis and induces down-regulation of PGC1α and its downstream targets, including TRB3, hypothetically leading to enhanced Akt phosphorylation levels. Secretion of TAG-enriched VLDL-1-type particles is decreased upon insulin stimulus.

Our data on VLDL secretion and glucose uptake suggests that both insulin and PPARγ might mediate some of their functions via affecting the expression of *ANGPTL3*. Activation of PPARγ decreases the transcription of insulin-responsive genes involved in the control of glucose production (gluconeogenesis) [34] and PPARγ can directly activate GLUT2 transporter thus influencing hepatic glucose uptake [35] which can explain the increased short-term glucose uptake levels upon rosiglitazone treatment in the present study. We show that silencing of *ANGPTL3* resulted in significant down-regulation of transcription factor *PGC1α* expression and its downstream targets *PEPCK* and *TRB3* (tribbles homologue 3), a protein kinase acting as a negative inhibitor for Akt phosphorylation [36,37]. Elevation of *PGC1α* expression in liver has been linked to IR in several mouse and human studies [37–39] thus the down-regulation of *PGC1α* and its downstream targets as observed in *ANGPTL3*-silenced cells suggest decreased levels of gluconeogenesis and fatty acid oxidation, processes which display aberrant activation in insulin resistant states (See Figure 9 as a summary for the pathways).

Insulin induces glucose uptake in insulin-responsive tissues (muscle, white adipose tissue) via GLUT4 but is not required for GLUT2-mediated glucose uptake by the liver [39]. In fact, insulin dose dependently decreases the expression of hepatocyte *GLUT2* during the first 6–12 h of insulin stimulation *in vitro* and *in vivo*. The inhibitory effect of insulin on *GLUT2* expression is abolished after 24 h despite continuous insulin stimulation and can also be reversed by high (10–20 mM) glucose concentration [21]. Therefore hepatic glucose uptake is driven by extracellular glucose concentration and not directly due to the elevated insulin levels (in other words GLUT2 is not insulin dependent). Increased glucose uptake in *ANGPTL3*-silenced cells might be due to higher production rate of GLUT2 and/or increased plasma membrane translocation and activation of GLUT2. In conclusion, our results give insights into the mechanisms of *ANGPTL3*-silencing in hepatic lipid and glucose metabolism. Since humans with ANGPTL3-deficiency display hypolipidaemia and insulin sensitivity it might be beneficial to target *ANGPTL3 i*n the liver, the major site of *ANGPTL3* expression, to balance lipid and glucose homoeostasis and lower the risk of cardiometabolic disorders.
